# Biocompatible Cobalt Oxide Nanoparticles for X-ray Fluorescence Microscopy

**DOI:** 10.21203/rs.3.rs-4312367/v1

**Published:** 2024-06-03

**Authors:** Christian Scott, Sophia Miller, Pierre Moenne-Loccoz, Craig Barnes, Martina Ralle

**Affiliations:** University of Tennessee at Knoxville; Oregon Health & Science University; Oregon Health & Science University; University of Tennessee at Knoxville; Oregon Health & Science University

**Keywords:** cobalt oxide nanoparticles, biocompatible, stealth coating, X-ray fluorescence microscopy, bioorthogonal chemistry, click chemistry

## Abstract

The synthesis of water-soluble nanoparticles is a well-developed field for ferrite-based nanoparticles with the majority consisting of iron oxide or mixed metal iron oxide nanoparticles. However, the synthesis of non-agglomerated non-ferrite metal/metal oxide NPs is not as well established. The synthesis and characterization of uniform 20 nm, biologically compatible cobalt oxide (CoO) nanoparticles (NPs) is described. These nanoparticles have two principle components: 1) a CoO core of suitable size to contain enough cobalt atoms to be visualized by X-ray fluorescence microscopy (XFM) and 2) a robust coating that inhibits NP aggregation as well as renders them water-soluble and biocompatible (i.e. stealth coatings). Stable cobalt oxide NPs are obtained with octadecyl amine coatings as reported by Bhattacharjee. Two strategies for solubilizing these NPs in water were investigated with varying degrees of success. Exchanging the octadecyl amine coating for a nitrodopamine anchored PEG coating yielded the desired water-soluble NPs but in very low yield. Alternately, leaving the octadecyl amine coating on the NP and interdigitating this with a maleic anhydride-vinyl copolymer with different hydrophobic sidechains followed by opening the maleic anhydride ring with amine substituted PEG polymers (the water solubilizing component), yielded the desired water soluble NPS were obtained in good yield. Characterization data for the nanoparticles and the components of the coatings required for bioorthogonal reactions to ligate them with biotargeting agents are also described.

## Introduction

The applications of metal-based nanoparticles are expanding rapidly as their synthesis and properties become better understood.[1, 2] One area of application involves utilization of nanoparticles conjugated with biotargeting agents to perform correlative imaging such as electron microscopy or synchrotron-based X-ray fluorescence microscopy (XFM). XFM has emerged as a powerful tool to quantitatively image trace elemental distributions in biological specimen.[3] Currently, beamlines at synchrotrons offer spatial resolutions ranging from the tens of microns to fifty nanometers and beyond. Sensitivity and resolution of XFM measurements are sufficient to distinguish cell types and organelles, but straightforward tools to identify cells and organelles simultaneously within one XFM scan are lacking. This technical in the in the interpretion of XFM data is expected to be further exacerbated by furture upgrades of synchrotron sources.

Several correlative approaches have been developed to match metal distributions to their precise location in different materials. One of these uses gold (Au) nanoparticles that are conjugated to 2° antibodies which in turn bind to 1° antibodies[4, 5]. XFM detection of the Au L-line fluorescence is then used to indirectly localize the proteins of interest and match it to the X-ray fluorescent signature of the desired element(s). However, Au X-ray fluorescence requires excitation energies above 10 keV (Au L-electron binding energies > 11,900 eV [6]), where beamline instrumentation optimized for biological specimen is much less efficient. Moreover, the significant overlap of the Zn K_β_ and Au L_α1_ emission lines can make imaging of Zn distributions difficult.[5]

The synthesis of NPs containing first row transition metal atoms is dominated by those containing iron in the form of iron oxide-based particles or containing endogenous metals such as copper, zinc or manganese.[7, 8] Because of the ubiquitous occurrence of iron in many biological systems we could not avail ourselves of iron or Fe-Co oxide-based NPs. Therefore, cobalt nanoparticles are ideal for XFM imaging in biological specimens because only trace amounts of endogenous cobalt are present, and the cobalt K_α_ emission line falls well within the ~ 10 keV excitation window and doesn’t show any significant overlap with other biologically relevant elements. We therefore sought to develop an efficient synthesis of biologically compatible, cobalt-based nanoparticles.

**Figure 1:**
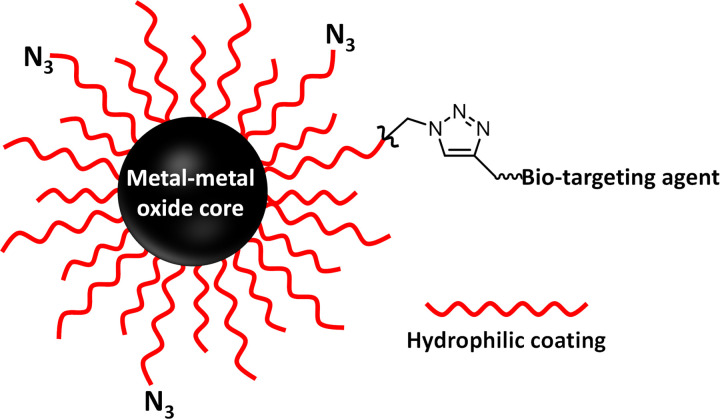
Illustration of a nanoparticle metal-metal oxide X-ray fluorescent spectroscopic probe biocompatible nanoparticle. The nanoparticle must be stabilized and made biocompatible via a robust coating of polymer molecules such as PEG chains. Conjugation with biotargeting agents such as antibodies, nanobodies or proteins is made possible by including functionalized PEG chains in the coating, in this case azide groups for bioorthoganol click reactions.

Beyond simple biocompatibility, it must also be possible to ligate such NPs with biotargeting molecules and other spectroscopic tags to study the actions of the NPs both *in vivo* and *in vitro* experiments. This latter goal is frequently accomplished by inserting chemical groups into the nanoparticle coating for bioorthogonal coupling strategies such as click reactions.[9] Here, we incorporated azidoe groups into our NP coating as shown in Fig. 1.

A few syntheses of cobalt and mixed metal iron-cobalt oxide nanoparticles have been reported [10–12] but, to our knowledge, none for cobalt alone with coatings that make them water soluble, nonaggregated, and tailored to inhibit immune responses in living systems. Furthermore, most pure cobalt oxide NP syntheses yield small (5–10 nm) NPs which would not contain enough cobalt to produce a detectable fluorescence signal for typical XFM conditions.[13]

## Methods and Results

**Figure 2:**
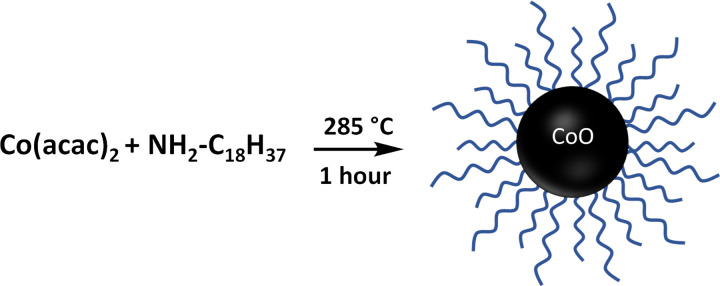
Synthesis of the initial hydrophobic cobalt oxide nanoparticle. The polymer coating is composed of octadecyl amine molecules.

Recently, Purkayastha and coworkers described a simple, high yield synthesis of unaggregated, CoO NPs using octadecylamine as both a solvent and surfactant coating (Fig. 2).[14] The size distribution for these hydrophobic nanoparticles was reported to be centered around 20 nm, which is ideal for XFM visualization applications. The identity and phase purity of the cobalt oxide-based NPs was verified by PXRD. Both PXRD and TEM data indicate that the NP size distribution is centered around 18–20 nm (electronic material).

**Figure 3:**
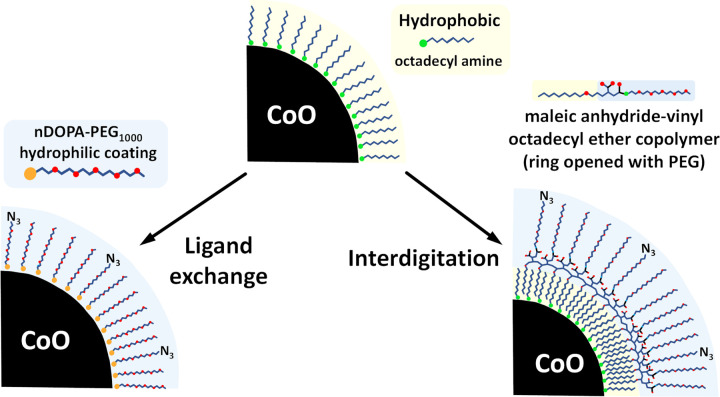
Two strategies for transforming initially synthesized hydrophobic NPs into biocompatible hydrophilic nanoparticles. The ligand exchange strategy involves replacing the initial hydrophobic coating while the interdigitating approach involves developing a bilayer-like arrangement with an outer hydrophilic component (PEG) making the nanoparticles water soluble.

The hydrophobic octadecyl amine coating renders these initial nanoparticles completely water insoluble. Two strategies were investigated to make them water soluble: 1) exchanging the amine-anchored coating with a water solubilizing polyethylene (PEG)-based coating having a nitro dopamine (nDOPA) anchoring group, and 2) leaving the amine-anchored coating in place and adding a bipolar copolymer that interdigitates with the octadecyl chains while providing a hydrophilic outer layer to solubilize the particles (Fig. 3).

**Figure 4:**
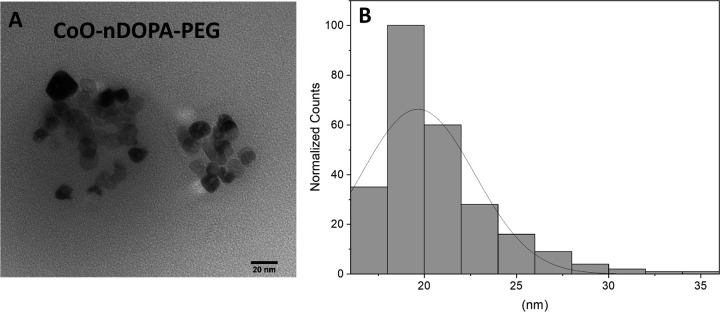
TEM image (**A**) and particle size distribution plot (**B**) derived from single particle ICPMS measurements of water-soluble CoO-nDOPA-PEG nanoparticle samples. Particle size distributions from both measurements maximized at 18 – 20 nm.

For the ligand exchange strategy, we chose the nitro dopamine (nDOPA) group to anchor the new PEG-based coating to the cobalt oxide surface due to its reported wide range of pH tolerance and nearly irreversible binding to oxide surfaces.[15, 16] Sonication of the hydrophobic nanoparticles with an excess of nDOPA-PEG_1000_-OMe (50–90%) and nDOPA-PEG_2000_-N_3_ (50 − 10%) followed by extraction into water was successful in creating water soluble nanoparticles but in very low yields (1–5%). The final, water soluble, PEG coated CoO nanoparticles were analyzed by cobalt elemental analysis (ICP-OES/MS), single particle ICPMS (spICPMS), PXRD, and TEM (Fig. 4) (see experimental details in EM to this Letter). Although the synthesis is direct (one step) and leads to very stable (months in aqueous solution) hydrophilic NPs, the very low yields in the ligand exchange step led us to search for higher yielding synthetic strategies.

**Figure 5:**
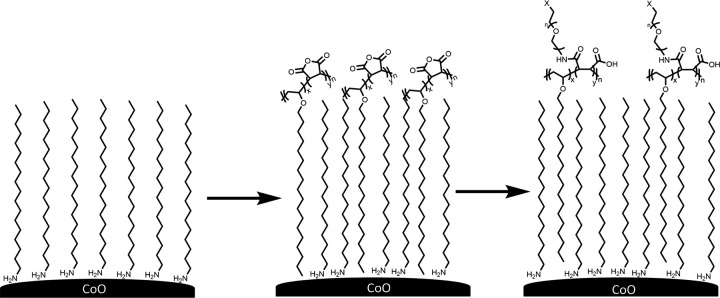
The interdigitation strategy leaves the initial octadecyl amine coating on the NP in place and involves two steps: First, exposure to the maleic anhydride-vinyl copolymer with a long hydrophobic sidechain causes the two hydrophobic polymers to mix (interdigitate) leaving the maleic anhydride ring exposed. In a second step, reaction with an amine functionalized PEG molecule opens the ring producing a hydrophilic outer coating containing both carboxylic acid groups and PEG chains. This outer coating renders the nanoparticles water soluble and biocompatible.

Schieber and coworkers recently described an interdigitation strategy for producing hydrophilic azide-modified CdSe/ZnS Core–shell quantum dots. [17] This interdigitation strategy involves two steps (Fig. 5). First, the C_18_NH_2_-coated NPs are exposed to a commercially available vinyl-maleic anhydride copolymer. The hydrophobic sidechains (phenyl, isobutylene, or octadecyl) of the co-polymer interdigitate or intertwine with the existing octadecyl amine coating developing a double coated nanoparticle. In a second *in situ* step, the maleic anhydride is ring-opened with an appropriately functionalized short-chain PEG polymer thus creating the desired, outer solubilizing, hydrophilic coating. The final product is extracted into water and purified by filtering through a 100k molecular weight centrifugation filter (Spin-X).

Three commercially available maleic anhydride copolymers were tested as well as several different amine PEG polymers (ethanol amine, H_2_N-PEG_150_-OMe, and H_2_N-PEG_550_-OMe) to determine the optimum combination that produced the desired water-soluble nanoparticles. No combination of PEG-amine and either the maleic anhydride-co-styrene or -co-isobutylene polymers produced water-soluble NPs. Only the combination of maleic anhydride-co-octadecyl vinyl ether (mal-C_18_) successfully interdigitated with the octadecylamine NP coating, and ultimately produced water soluble nanoparticles.

After opening of the maleimide ring with ethanol amine and H_2_N-PEG_150_-OMe only very low amounts of water-soluble NPs were obtained. However, the longer PEG amine (H_2_N-PEG_550_-OMe) succeeded in producing much more concentrated aqueous solutions of nanoparticles. This combination gave a desired water-soluble cobalt oxide nanoparticle product in good yield (50% wt).

**Figure 6:**
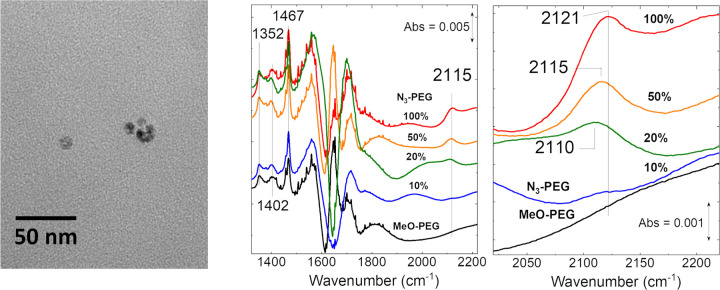
Right: TEM image the C18 interdigitated cobalt oxide nanoparticles. Left: Transmittance IR data for aqueous solutions of different PEG-azide loadings in the outer coating of the interdigitated nanoparticles. The azide stretch can be observed down to ~20% loading in the PEG outer coating around each nanoparticle.

TEM images of the water-soluble interdigitated NPs showed that the sizes of the cobalt oxide cores of the NPs had not significantly changed (< *d* > = 20 nm; Fig. 6). Transmittance IR spectra of aqueous solutions of the NPs with different amounts of the PEG-azide in their coating were obtained. Clear evidence for the presence of the azide group was observed (N_3_ stretch at ~ 2110–2120 cm^− 1^) down to 20% loading of the PEG-azide with the bulk of the coating made up of PEG-OMe chains.

## Discussion-Nanoparticle Stability

The shelf stability of the ligand exchange NPs and interdigitation NPs is on the order of months with no visual evidence of aggregation leading to precipitation of the NPs. Additionally, nanoparticle batches may be dried and resuspended with sonication with no visual signs of aggregation. This long-term stability shows that both types of nanoparticles are quite stable and can be prepared and stored for long periods of time.

## Summary and Future Work

The successful synthesis of functionalized cobalt oxide nanoparticles through two pathways provides a convenient framework with which to further elaborate these NPs for use as bio-applicable XFM probes. The azide groups terminating PEG chains in the outer coatings around the NPs are often used for this purpose via bioorthogonal click reactions. Current work is now focused on characterizing different nanoparticle-biotargeting agent constructs (e.g. antibodies, nanobodies) and developing protocols for *in vitro* and *in vivo* X-ray fluorescence microscopy experiments.

## Supplementary Material

Supplement 1

## Data Availability

All data is provided in the electronic supplementary material in addition to their corresponding references
