# Supplementary material for: Biocompatible Cobalt Oxide Nanoparticles for X-ray Fluorescence Microscopy
Source: Res Sq. 2024 Jun 3:rs.3.rs-4312367. Preprint. [Version 1] doi: 10.21203/rs.3.rs-4312367/v1 (PMC11177975; doi:10.21203/rs.3.rs-4312367/v1)
Supplement: Supplement 1 [file NIHPPrs4312367v1-supplement-1.pdf]

## Supplementary Files

This is a list of supplementary files associated with this preprint. Click to download.

- BiocompatibleCobaltOxideNanoparticlesforXrayFluorescenceMicroscopyEM.docx
